# Effects of Light–Nitrogen Interactions on Leaf Functional Traits of (*Picea neoveitchii* Mast.)

**DOI:** 10.3390/plants14162550

**Published:** 2025-08-16

**Authors:** Sibo Chen, Siyu Yang, Wanting Liu, Kaiyuan Li, Ninghan Xue, Wenli Ji

**Affiliations:** College of Landscape Architecture and Arts, Northwest A&F University, Xianyang 712100, China; chensibo@nwafu.edu.cn (S.C.); byyangsiyu@163.com (S.Y.);

**Keywords:** light–nitrogen interaction, leaf functional traits, photosynthesis, stress resistance, ecological adaptation

## Abstract

*Picea neoveitchii* Mast., a critically endangered spruce species endemic to China, is classified as a national second-level key protected wild plant and listed as critically endangered (CR) on the International Union for Conservation of Nature (IUCN) Red List. Its habitat features complex forest light environments, and global climate change coupled with environmental pollution has increased regional nitrogen deposition, posing significant challenges to its survival. This study explores the effects of light–nitrogen interactions on the leaf functional traits of *Picea neoveitchii* Mast. seedlings by simulating combinations of light intensities (100%, 70%, and 40% full sunlight) and nitrogen application levels (0, 10, and 20 g N·m ^−2^·a^−1^, where g N·m^−2^·a^−1^ denotes grams of nitrogen applied per square meter per year). We examined changes in morphological traits, anatomical structures, photosynthetic physiology, and stress resistance traits. Results indicate that moderate shading (70% full sunlight) significantly enhances leaf morphological traits (e.g., leaf length, leaf area, and specific leaf area) and anatomical features (e.g., mesophyll tissue area and resin duct cavity area), improving light capture and stress resistance. Medium- to high-nitrogen treatments (10 or 20 g N·m^−2^·a^−1^) under moderate shading further increase photosynthetic efficiency, stomatal conductance, and antioxidant enzyme activity. According to the comprehensive membership function evaluation, the L2N0 (70% full sunlight, 0 g N·m^−2^·a^−1^) treatment exhibits the most balanced performance across both growth and stress-related traits. These findings underscore the critical role of light–nitrogen interactions in the growth and adaptability of *Picea neoveitchii* Mast. leaves, offering a scientific foundation for the conservation and ecological restoration of endangered plant populations.

## 1. Introduction

*Picea neoveitchii* Mast., a national second-level key protected wild plant endemic to China, is listed by the National Forestry and Grassland Administration of China under the “Wild Plants with Extremely Small Population (WPESP)” directory and classified as critically endangered (CR) on the International Union for Conservation of Nature (IUCN) Red List of Threatened Species [1,2]. This species is primarily distributed in the Qinling Mountains and their subsidiary ranges, thriving in specific habitats such as valley bottoms or semi-shaded slopes within mixed coniferous–broadleaf forests, where it exhibits stringent ecological requirements [3]. In recent years, overlogging and habitat loss have precipitated a drastic population decline, garnering widespread concern for its survival. Current research has predominantly explored endangerment mechanisms, community characteristics, genetic diversity, and seedling cultivation techniques, revealing a significant population decline driven by habitat fragmentation, weak sexual reproduction, high seed emptiness rates, and low germination success [4,5]. However, investigations into its physiological and ecological adaptation mechanisms remain nascent, particularly regarding response strategies to light–nitrogen interactions.

Light is the energy base that drives photosynthesis, and nitrogen is one of the essential nutrients that plants need most, and they coordinate the allocation strategy of plant resources [6,7,8]. Light fuels photosynthesis by converting solar energy into chemical energy, underpinning organic matter synthesis and energy flow within the biosphere [9]. A plant’s capacity to adapt to light conditions shapes its competitive and survival potential within ecosystems, with light intensity, quality, and photoperiod influencing photosynthetic efficiency, growth morphology, and metabolic regulation [9,10,11]. Optimal light enhances photosynthetic rates and growth, whereas insufficient or excessive light induces photosynthetic inhibition, metabolic disruption, and growth limitation [12]. Nitrogen, an essential nutrient, forms the backbone of nucleic acids, proteins, and chlorophyll, playing a critical role in plant growth, metabolism, and reproduction [13,14]. Its availability directly impacts photosynthesis, carbon–nitrogen metabolism, and biomass accumulation, while shifts in soil nitrogen levels reflect adaptive strategies to environmental nutrient dynamics [15]. Amid global climate change and escalating environmental pollution, heightened regional nitrogen deposition profoundly alters plant community structure and species diversity, underscoring the ecological imperative of elucidating light and nitrogen effects on plants [16].

Plant functional traits underpin their adaptation to environmental conditions and life processes, spanning morphological, physiological, and ecological dimensions [17]. As the primary interface with the environment, leaves serve as hubs for photosynthesis, transpiration, and respiration, rendering their functional traits highly responsive to environmental shifts and valuable for studying adaptability [18]. These traits encompass macroscopic morphology (e.g., leaf area, specific leaf area, and dry matter content), which indicate resource acquisition and utilization efficiency and reflect growth strategies; microscopic anatomy (e.g., stomatal density and tissue structure), which governs transpiration and photosynthetic efficiency; and biochemical properties (e.g., chlorophyll content), which signal photosynthetic capacity and nutrient status [9,19,20,21]. Nitrogen (N) and phosphorus (P) are fundamental to plant physiology: N drives photosynthesis, metabolism, nutrient storage, and development, while P supports nucleic acids and phospholipids critical for growth and reproduction [22,23]. The N/P ratio illuminates nutrient uptake efficiency and growth rates [24]. Recent research increasingly emphasizes leaf photosynthetic traits and stress resistance, employing parameters such as net photosynthetic rate (Pn) and stomatal conductance (Gs) to assess adaptability across conditions, chlorophyll fluorescence to non-destructively probe photosystem dynamics, and metabolic regulation (e.g., protective enzymes, osmolytes, and non-structural carbohydrates) to explore stress responses [25,26,27,28].

This study targets *Picea neoveitchii* Mast. to elucidate its adaptation strategies to heterogeneous environments by examining leaf functional trait responses under varying light and nitrogen conditions. Through controlled simulation experiments, we aim to delineate the pathways by which light–nitrogen interactions influence its growth and physiological metabolism, specifically addressing how leaf traits respond to light intensity and soil nitrogen gradients. Set against the backdrop of global climate change and rising nitrogen deposition, this research seeks to unravel the effects of light–nitrogen interplay on *Picea neoveitchii* Mast.’s growth and adaptability. By enriching theoretical frameworks for ecological adaptation in endangered plants, our findings aim to provide a scientific foundation for the conservation and restoration of this critically imperiled species.

## 2. Results

### 2.1. Effects of Light–Nitrogen Interaction on Needle Morphological and Anatomical Traits of Picea neoveitchii Mast. Seedlings

**Figure 1:**
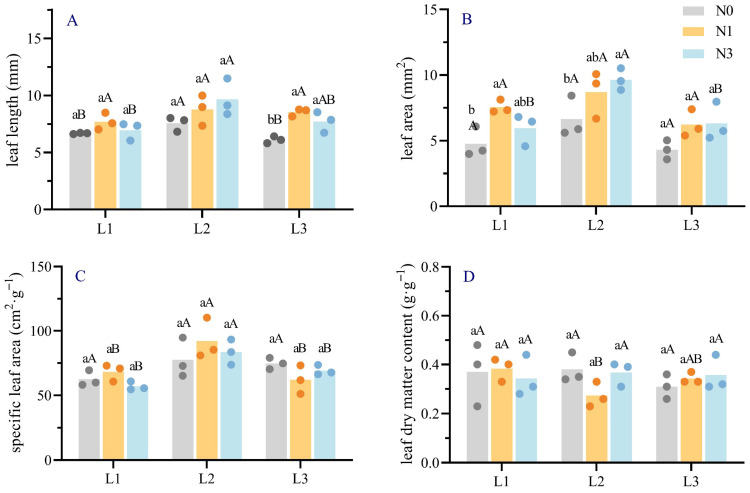
Effects of light and nitrogen interaction on morphological characteristics of needles of *Picea neoveitchii* Mast. seedlings. (L1: 100% full light, L2: 70% full light, and L3: 40% full light; N0: 0 g N m^−2^·a^−1^, N0: 10 g N m^−2^·a^−1^, and N1: 20 g N m^−2^·a^−1^. Different lowercase letters indicate significant differences (*p* < 0.05) between different nitrogen application groups under the same shading treatment; different capital letters indicate significant differences (*p* < 0.05) between different shading treatment groups under the same nitrogen application treatment). (**A**) is leaf length, (**B**) is leaf area, (**C**) is specific leaf area, and **D** is leaf dry matter content.

Significant differences were observed in needle length, leaf area, specific leaf area (SLA), and leaf dry matter content of *Picea neoveitchii* Mast. seedlings under varying shading and nitrogen treatments (Figure 1). Needle length was highest in the L2N2 treatment (9.66 mm), 1.45 times greater than the control group L1N0 (CK, the following have been referred to by CK, 6.67 mm). Under identical light conditions, needle length showed a non-significant variation across nitrogen levels in the L1 treatment, while in L3, only the N0 treatment resulted in a significantly longer needle length compared to N1 and N2. In contrast, needle length consistently increased with nitrogen addition under the L2 treatment. Under identical nitrogen levels, decreasing light intensity resulted in similar trends in needle length. Leaf area was greatest in the L2N2 treatment (9.64 mm^2^), 2.02 times that of the control. In L1 treatments, leaf area initially increased with nitrogen application and then decreased, whereas it consistently increased in L2 and L3 treatments. SLA was highest in the L2N1 treatment (92.17 cm^2^·g^−1^), significantly exceeding other treatments. Leaf dry matter content showed minimal variation, with the highest values in L1N1 and L2N0 treatments (0.38 g·g^−1^), slightly above the control (0.37 g·g^−1^).

Among needle traits (Table 1), needle thickness was greatest in the L2N0 treatment, 1.46 times that of L2N2. Stomatal density was highest in the CK treatment, 2.41 times that of L2N0. Under the L2 treatment, stomatal density showed an increasing trend with nitrogen addition, and the N2 treatment exhibited a significantly higher value than N0. In contrast, under L3, no consistent or significant pattern was observed in stomatal density across nitrogen levels. Needle cross-sectional area and perimeter reached their maximum values in the L2N0 treatment, but the differences among nitrogen treatments were not statistically significant in some light conditions. Epidermal tissue area and mesophyll tissue area were greatest in the L2N0 treatment, decreasing with increasing nitrogen application. Resin duct cavity area was largest in the L3N0 treatment, 4.75 times that of CK and L2N2, with notable variation across nitrogen levels. Endodermal and central cylinder areas were greatest in the L2N0 treatment, decreasing with increasing nitrogen application. The proportions of mesophyll and resin duct cavity areas were highest in the L3N0 treatment, exhibiting distinct variation trends.

### 2.2. Effects of Light–Nitrogen Interaction on N and P Stoichiometric Characteristics of Picea neoveitchii Mast. Seedling Needles

**Figure 2:**
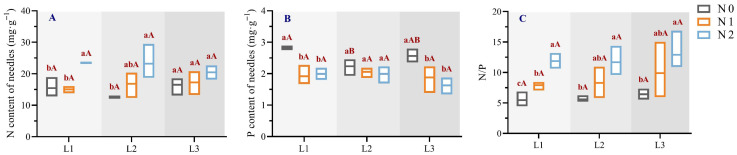
Effects of light–nitrogen interactions on the stoichiometric characteristics of N and P in needles of *Picea neoveitchii* Mast. seedlings. (L1: 100% full light, L2: 70% full light, and L3: 40% full light; N0: 0 g N m^−2^·a^−1^, N1: 10 g N m^−2^·a^−1^, and N2: 20 g N m^−2^·a^−1^. Different lowercase letters indicate significant differences (*p* < 0.05) between different nitrogen application groups under the same shading treatment; different capital letters indicate significant differences (*p* < 0.05) between different shading treatment groups under the same nitrogen application treatment). (**A**) is N content of needles, (**B**) is P content of needles, and (**C**) is N/P of needles.

Under varying shading and nitrogen treatments (Figure 2), nitrogen content was highest in the L1N2 treatment (23.49 mg·g^−1^), 1.89 times that of the lowest value in the L2N0 treatment. Phosphorus content was highest in the CK treatment (2.83 mg·g^−1^), 1.74 times that of the lowest value in the L3N2 treatment. The N/P ratio was highest in the L3N2 treatment (12.90), 2.35 times that of the control. Under identical light conditions, nitrogen content did not show statistically significant differences among nitrogen treatments in L1, L2, or L3 groups, although a slight increase was observed in L2 and L3. Phosphorus content also exhibited no significant differences among nitrogen levels, and no consistent pattern could be established across light treatments. The N/P ratio significantly increased with higher nitrogen application. Under identical nitrogen levels, nitrogen and phosphorus contents exhibited distinct patterns with changing light intensity, while the N/P ratio generally increased with decreasing light intensity across most treatments.

### 2.3. Effects of Light–Nitrogen Interaction on Photosynthetic Physiological Traits of Picea neoveitchii Mast. Seedling Needles

Under varying shading and nitrogen treatments (Table 2), chlorophyll a content was highest in the L3N2 treatment (0.261 mg·g^−1^), 2.06 times that of the L3N1 treatment. The chlorophyll content in N1 decreased compared to the control (only in L2, the decrease was insignificant), while a significant increase under the influence of nitrogen was only present in L1N2. Chlorophyll b content was highest in the L3N2 treatment (0.205 mg·g^−1^); it decreased with increasing nitrogen in the L1 treatments, whereas in the L2 and L3 treatments, the highest values occurred in N2. Total chlorophyll content was greatest in the L3N2 treatment (0.465 mg·g^−1^), decreasing with increasing nitrogen in the L1 treatments, but showing a decline followed by an increase in the L2 and L3 treatments. Carotenoid content showed no significant differences; in the L1 and L2 treatments, it initially decreased then increased with nitrogen application, while in the L3 treatments, it consistently decreased.

**Figure 3:**
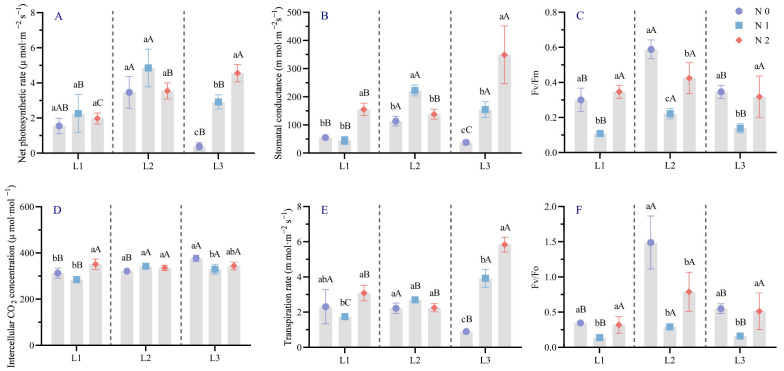
Effects of light and nitrogen interaction on photosynthetic parameters and chlorophyll fluorescence parameters of *Picea neoveitchii* Mast. seedlings. (L1: 100% full light, L2: 70% full light, and L3: 40% full light; N0: 0 g N m^−2^·a^−1^, N1: 10 g N m^−2^·a^−1^, and N2: 20 g N m^−2^·a^−1^.) (**A**) is net photosynthetic rate, (**B**) is Stomatal conductance, (**C**) is Fv/Fm, (**D**) is intercellular CO_2_ concen-tration, (**E**) is transpiration rate, (**F**) is Fv/Fo. Different lowercase letters indicate significant differences (*p* < 0.05) between different nitrogen application groups under the same shading treatment; different capital letters indicate significant differences (*p* < 0.05) between different shading treatment groups under the same nitrogen application treatment).

Analysis of photosynthetic and chlorophyll fluorescence parameters of *Picea neoveitchii* Mast. seedling needles (Figure 3) revealed that the net photosynthetic rate was highest in the L2N1 treatment (4.85 μmol·m^−2^·s^−1^), 3.13 times that of the control group. In the L1 and L2 treatments, net photosynthetic rate showed no statistically significant differences among nitrogen levels, although slight fluctuations in mean values were observed. In the L3 treatments, a modest increase in net photosynthetic rate was observed with increasing nitrogen application, but this trend was not statistically significant. Stomatal conductance was highest in the L3N2 treatment (348.92 mmol·m^−2^·s^−1^), 6.33 times that of the control, with varied trends across nitrogen levels. Intercellular CO_2_ concentration peaked in the L3N0 treatment (376.58 μmol·mol^−1^), and transpiration rate was highest in the L3N2 treatment (5.84 mmol·m^−2^·s^−1^). Water use efficiency and transpiration rate exhibited diverse trends with changes in light and nitrogen levels. For chlorophyll fluorescence parameters, both Fv/Fm and Fv/Fo were highest in the L2N0 treatment (0.589 and 1.489, respectively), 1.96 and 3.33 times those of the control. Under identical light conditions, these parameters generally decreased then increased with increasing nitrogen application; under identical nitrogen levels, they typically increased then decreased with decreasing light intensity.

### 2.4. Effects of Light–Nitrogen Interaction on Stress Resistance Physiological Traits of Picea neoveitchii Mast. Seedling Needles

**Figure 4:**
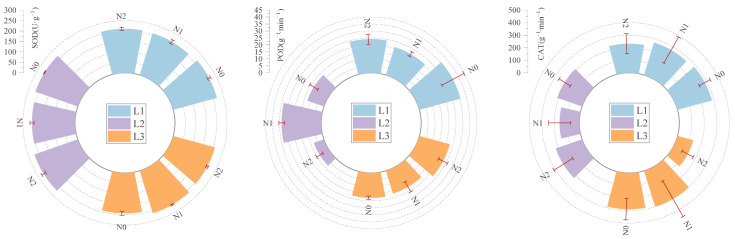
Effects of light and nitrogen interaction on needle protective enzyme activity of *Picea neoveitchii* Mast. seedlings. (L1: 100% full light, L2: 70% full light, and L3: 40% full light; N0: 0 g N m^−2^·a^−1^, N1: 10 g N m^−2^·a^−1^, and N2: 20 g N m^−2^·a^−1^. Error bars represent standard deviation. Significance was assessed at *p* < 0.05).

Under varying shading and nitrogen treatments (Figure 4), superoxide dismutase (SOD) activity peaked in the L3N2 treatment at 220.03 U·g^−1^, 1.01 times that of the control group. Under identical light conditions, SOD activity trends varied with nitrogen application, showing patterns of initial decline followed by an increase or vice versa depending on light intensity. Peroxidase (POD) activity was highest in the control group CK (30.59 U·g^−1^·min^−1^), four times that of the lowest value in L2N2, exhibiting complex interactions with nitrogen application and shading levels. Catalase (CAT) activity showed no significant differences, with the highest value in the L3N1 treatment (299.25 U·g^−1^·min^−1^), 1.06 times that of the control. CAT activity displayed varied trends across light and nitrogen levels, such as decreasing then increasing or increasing then decreasing with nitrogen application.

Under varying shading and nitrogen treatments (Table 3), the trends of multiple physiological indicators of the needles of *Picea neoveitchii* Mast. seedlings were different. The highest soluble protein content was observed in the L2N2 treatment (1.73 mg·g^−1^), though not significantly different from the control (CK). Free proline content was highest in the L1N2 treatment (445.05 μg·g^−1^), 1.46 times that of the control. Malondialdehyde (MDA) content was highest in the CK treatment (1.396 mmol·g^−1^), 3.08 times that of the lowest value in L1N1. Relative conductivity reached its maximum in the CK treatment (73.42%), 1.98 times that of L3N1. Soluble sugar content was highest in the L2N0 treatment (70.50 mg·g^−1^), while needle starch content peaked in the L1N2 treatment (13.92 mg·g^−1^). Additionally, non-structural carbohydrate (NSC) content was highest in the L2N0 treatment (82.03 mg·g^−1^), significantly exceeding other treatments. Overall, while some indicators like MDA and relative conductivity consistently decreased under shading and nitrogen treatments, others such as starch and NSC exhibited treatment-specific responses without a clear directional trend.

### 2.5. Membership Function Analysis of Leaf Functional Traits of Picea neoveitchii Mast. Seedlings

The effects of light and nitrogen on leaf functional traits of *Picea neoveitchii* Mast. seedlings are complex, resulting from multifaceted interactions. This study employed membership function analysis to comprehensively evaluate leaf functional traits under different shading and nitrogen treatments. Based on membership values (U), environmental adaptability was classified into four levels: non-resistant (U < 0.2), weakly resistant (0.2 ≤ U < 0.4), moderately resistant (0.4 ≤ U < 0.6), and highly resistant (0.6 ≤ U ≤ 1). As shown in Table 4, the mean membership values ranked as L2N0 > L2N2 > L2N1 > L1N2 > L3N0 > L3N2 > CK > L1N1 > L3N1, with L2N0 exhibiting the highest value (0.592) and L3N1 the lowest (0.362), ranging from 0.362 to 0.592. This indicates enhanced stress resistance under optimal light and nitrogen conditions. According to the principle that higher membership values correlate with greater growth promotion, treatments L2N0, L2N2, L2N1, L1N2, L3N0, and L3N2 promoted seedling growth to varying degrees compared to the control (CK). Among the treatments, L2N0 (70% full sunlight, no nitrogen) exhibited the highest comprehensive membership score (0.592), indicating a comparatively strong performance in promoting seedling growth, followed by L2N2. This suggests that, even without nitrogen application, 70% full sunlight enhanced seedling morphology, physiology, and stress resistance. Medium- to high-nitrogen treatments (N1, N2) under moderate shading (L2) further improved adaptability, particularly by increasing photosynthetic efficiency and antioxidant capacity. In contrast, low light intensity (L3) significantly reduced adaptability, likely due to limited photosynthetic carbon assimilation and energy acquisition, with high nitrogen exacerbating carbon–nitrogen imbalances under low light. Thus, moderate shading and nitrogen application are key ecological strategies for optimizing *Picea neoveitchii* Mast. seedling growth.

## 3. Discussion

### 3.1. Effects of Light–Nitrogen Interaction on Needle Morphological Traits of Picea neoveitchii Mast. Seedlings

Light is the energy base that drives photosynthesis, and nitrogen is one of the essential nutrients that plants need most, and they coordinate in the allocation of plant resources [29]. This study demonstrates that needle length, leaf area, and specific leaf area (SLA) of *Picea neoveitchii* Mast. seedlings peaked under moderate shading (L2), with significant increases under medium- to high-nitrogen treatments (N1, N2) (Figure 1). Given the slow growth rate of *Picea neoveitchii* Mast. and the influence of needle cohort phenology, the responses observed here likely represent short-term acclimation mechanisms rather than long-term developmental adaptations. These changes in needle morphology reflect adaptive resource allocation strategies: under low light, increased needle length and SLA enhanced light capture to mitigate photosynthetic limitations, consistent with mechanisms proposed by Jerzy Modrzyński et al. for low-light adaptation [30,31]. However, excessive shading (L3) significantly reduced leaf area and needle length, likely due to insufficient photosynthetic products to support leaf growth [32]. Nitrogen application significantly increased needle length, indicating its role in promoting cell division and elongation [33]. Yet, excessive nitrogen (N2) led to reduced leaf area and needle length under some light conditions, possibly due to soil acidification and nutrient imbalances inhibiting growth [34]. The significant interaction between shading and nitrogen treatments indicates that moderate light–nitrogen synergy is critical for optimizing needle morphology [35]. Regarding anatomical traits, the L2N0 treatment significantly increased needle cross-sectional area, perimeter, and mesophyll tissue area, enhancing light absorption and photosynthetic capacity (Table 1). The larger resin duct cavity area and proportion observed in L2N1 suggest enhanced structural defense under moderate-light and low-nitrogen conditions. This may be associated with the stimulation of secondary metabolite synthesis, reflecting the morphological and anatomical adaptations of *Picea neoveitchii* Mast. to combined light–nitrogen variation [36,37].

Nitrogen (N) and phosphorus (P) are vital for photosynthesis, energy metabolism, and growth, with the N/P ratio serving as an indicator of nutrient limitation [38]. This study found that shading had minimal impact on needle N and P stoichiometric characteristics (Figure 2). Under low nitrogen levels (N0, N1), low light (L3) increased needle nitrogen content, likely due to its enhanced accumulation to support protein, chlorophyll, and nucleic acid synthesis for sustained growth in low-light, low-nitrogen conditions [39]. The plant growth rate hypothesis suggests that relative growth rate correlates positively with phosphorus content and P/N ratio [40]. Here, needle phosphorus content decreased with reduced light intensity, and the N/P ratio was highest under low light (L3), indicating that shading constrained seedling growth rates, consistent with prior studies [41,42]. Nitrogen application significantly increased needle nitrogen content and N/P ratio but reduced phosphorus content, suggesting that excessive nitrogen may induce nutrient imbalances, exacerbating phosphorus limitations [43]. This may be attributed to nitrogen-induced suppression of phosphorus uptake, likely caused by nutrient antagonism, microbial competition, or altered root absorption capacity under excessive nitrogen. Needle N/P ratios ranged from 5.48 to 12.90, below the threshold of 14, indicating that there was nitrogen limitation resulting from all treatments for *Picea neoveitchii* Mast. seedlings. The high background level of available nitrogen may have partially offset the response to nitrogen addition, as also reflected in the limited physiological gains with high-nitrogen treatments.

### 3.2. Effects of Light–Nitrogen Interaction on Photosynthetic Physiological Traits of Picea neoveitchii Mast. Seedling Needles

Photosynthetic pigments and parameters are central indicators of plant growth and productivity [22]. In this study, chlorophyll a, chlorophyll b, and total chlorophyll contents of *Picea neoveitchii* Mast. seedlings peaked under low-light and high-nitrogen conditions (L3N2) (Table 2). In low-light environments, plants typically increase photosynthetic pigment content, particularly chlorophyll b, to enhance blue–violet light absorption [44], thereby improving adaptation to low light. The lowest chlorophyll a/b ratio being in the L3N2 treatment further indicates enhanced light utilization and adaptability under low light and high nitrogen, consistent with findings in shade-tolerant *Panax notoginseng*, where high nitrogen significantly increased photosynthetic rate and stomatal conductance, boosting light-use efficiency in dynamic light conditions [45]. Moreover, ample nitrogen supply promoted chlorophyll synthesis, further enhancing photosynthetic efficiency, which is particularly critical for plants in low-light environments [46].

Analysis of photosynthetic parameters revealed that the net photosynthetic rate (Pn) of *Picea neoveitchii* Mast. seedlings was highest under moderate shading (L2) and medium nitrogen (N1), significantly surpassing other treatments (Figure 3). Moderate shading mitigated photoinhibition from excessive light, while increased leaf area and specific leaf area enhanced photosynthetic efficiency [47]. Higher stomatal conductance (Gs) and transpiration rate (Tr) in L2 treatments further supported this, indicating that optimal light–nitrogen conditions improved needle structure, water transport, and photosynthetic capacity. However, under excessive shading (L3) or high nitrogen (N2), Pn and Gs significantly declined, likely due to damage to mesophyll cell structure and photosynthetic organelle function [48]. Higher nitrogen input (e.g., N2 treatment) may have increased needle nitrogen content and slightly altered photosynthetic pigment composition, but it did not lead to significant improvements in photosynthetic rate (Pn) or stomatal conductance (Gs). This suggests that beyond a certain threshold, additional nitrogen may not further enhance physiological function, and could potentially disrupt metabolic balance or interfere with stomatal regulation [49,50]. Chlorophyll fluorescence parameters (Fv/Fm and Fv/Fo) peaked in the L2N0 treatment, indicating that moderate shading and low nitrogen significantly enhanced PSII photochemical efficiency and light capture capacity. In contrast, these parameters decreased in the L3N2 treatment, suggesting that excessive shading and high nitrogen induced low-light stress and nutrient imbalance, impairing normal photosynthesis [51,52].

### 3.3. Effects of Light–Nitrogen Interaction on Stress Resistance Physiological Traits of Picea neoveitchii Mast. Seedling Needles

The stress resistance physiological traits of *Picea neoveitchii* Mast. seedling needles exhibited high sensitivity to light–nitrogen treatments, particularly in protective enzyme activity and osmoregulatory substances [53] (Figure 4). Superoxide dismutase (SOD) activity peaked in the L3N2 treatment, indicating that under low-light and high-nitrogen conditions, plants may have experienced elevated oxidative stress, which triggered enhanced antioxidant enzyme activity to mitigate reactive oxygen species (ROS) accumulation and protect cellular integrity [54]. Studies show that environmental stress induces accumulation of ROS, such as superoxide anions, hydroxyl radicals, and hydrogen peroxide, which can damage cell membranes and other components [55]. Plants counteract this by activating antioxidant enzyme systems to maintain cellular function [56]. However, peroxidase (POD) activity was highest in the control group (CK), while L2N2 significantly reduced POD activity. This response highlights a typical stress-adaptive mechanism, where reduced light may limit photosynthetic electron flow, but in combination with high nitrogen input, metabolic imbalance may still lead to ROS formation, thus requiring enhanced SOD-mediated defense. Excessive nitrogen, however, may cause metabolic imbalances, triggering oxidative stress [57,58]. Osmoregulatory substances, such as soluble protein and free proline, were significantly affected by light–nitrogen treatments [59,60]. Soluble protein content peaked in the L2N2 treatment, while free proline content was highest in L3N2, indicating enhanced stress resistance through osmoregulation under low light and high nitrogen [61]. Malondialdehyde (MDA) content and relative conductivity were highest in the control (L1N1), suggesting that moderate shading (L2) combined with low to moderate nitrogen application (particularly L2N1) effectively alleviated oxidative damage and improved membrane stability [62]. This aligns with the membership function results, where the L2N1 treatment showed the best integrated performance in terms of both growth and stress resistance. Moderate shading combined with nitrogen application activated antioxidant defense mechanisms, particularly by enhancing SOD, POD, and catalase (CAT) activities to scavenge excess ROS, protecting cell membranes, reducing lipid peroxidation, and enhancing membrane stability [63,64]. Although L2N0 had the highest comprehensive score based on membership function, it did not outperform in all individual traits. Thus, this result suggests a balanced performance across morphology, physiology, and resistance metrics rather than superiority in specific dimensions.

## 4. Materials and Methods

### 4.1. Study Area

This experiment was conducted at a forest farm in Wudu District, Longnan City, southeastern Gansu Province, China. Wudu District (104°34′–105°38′ E, 32°47′–33°42′ N) is located in the middle reaches of the Bailong River, a tributary of the Jialing River in the Yangtze River Basin, at the intersection of Gansu, Shaanxi, and Sichuan provinces within the Qinba Mountain Range. The terrain slopes from northwest to southeast, with elevations ranging from 667 to 3600 m [65]. The climate is transitional between a north subtropical humid and a warm temperate semi-arid zone, exhibiting distinct vertical zonation with subtropical, warm temperate, and cold temperate characteristics due to its complex topography. The region experiences dry springs and winters with minimal rain or snow, rainy autumns, and frequent heavy rain or hail from June to September. The annual average temperature is 14.9 °C, with 1872 h of sunshine, a frost-free period of 210–240 days, and approximately 460 mm of annual precipitation. Vegetation primarily consists of north subtropical evergreen and deciduous broadleaf mixed forests, with additional deciduous broadleaf forests and dark coniferous forests influenced by the variable mountainous climate [66]. The soil in the experimental area is weakly acidic (pH 6.85) with moderate levels of organic matter (12.713 g·kg^−1^) and total nitrogen (0.993 g·kg^−1^), indicating fertile conditions suitable for *Picea neoveitchii* Mast. growth.

### 4.2. Experimental Materials

The experimental seedlings were 9-year-old *Picea neoveitchii* Mast. provided by the Forestry Research Institute of the Bailongjiang Forestry Administration, Gansu Province, sourced from a native population in Zhouqu County, Gannan, Gansu. In November 2023, seedlings were transplanted into containers (50 cm in diameter and 50 cm in height), with one seedling per container. The containers were filled with topsoil collected from the Wudu District Forest Farm, which had the following properties: pH 6.85; total nitrogen, 0.993 g·kg^−1^; total phosphorus, 0.737 g·kg^−1^; total potassium, 10.429 g·kg^−1^; available nitrogen, 0.469 g·kg^−1^ (determined using the alkali hydrolysis diffusion method); available phosphorus, 8.459 mg·kg^−1^; available potassium, 85.472 mg·kg^−1^; and organic matter, 12.713 g·kg^−1^. Each seedling remained in its container throughout the experimental period. After acclimation until April 2024, healthy and uniform seedlings (mean height 35.98 cm, mean basal diameter 9.09 mm) were selected as experimental materials.

Prior to the experiment, the seedlings had been cultivated under partially shaded conditions at approximately 70% of full sunlight. During the 90-day experimental period (April to June 2024), local meteorological data recorded by the Wudu District Forestry Station indicated the following monthly averages: April: mean air temperature of 13.9 °C (daily high: 20.5 °C; daily low: 8.1 °C), average relative humidity of 57%, and mean solar radiation of 15,265 kJ·m^−2^·d^−1^; May: mean air temperature of 19.8 °C (daily high: 26.2 °C; daily low: 14.3 °C), relative humidity of 60%, and solar radiation of 17,374 kJ·m^−2^·d^−1^; and June: mean air temperature of 24.7 °C (daily high: 30.6 °C; daily low: 19.4 °C), relative humidity of 64%, and solar radiation of 18,330 kJ·m^−2^·d^−1^. In addition, environmental conditions during photosynthetic measurements—such as external photosynthetically active radiation (PAR_e_), ambient temperature, and relative humidity—are summarized in Supplementary Table S1.

### 4.3. Experimental Design

Shading and nitrogen application treatments were conducted from early April to early July 2024. Shading treatments included three levels: L1 (100% full sunlight), L2 (70% full sunlight), and L3 (40% full sunlight), achieved using shade nets with varying light transmittance. Nitrogen treatments comprised three levels: N0 (0 g N·m^−2^·a^−1^, where g N·m^−2^·a^−1^ denotes grams of nitrogen applied per square meter per year), N1 (10 g N·m^−2^·a^−1^), and N2 (20 g N·m^−2^·a^−1^). Nitrogen was applied as NH_4_NO_3_, calculated based on treatment levels, dissolved, and administered in two doses (early April and mid-May) of 200 mL per seedling per application. The experiment included eight treatment groups and one control group (L1N0, CK); each treatment group consisted of four replicates (*n* = 4), totaling 36 seedlings. During the treatment period, dedicated personnel managed irrigation and pest control. The treatment design is detailed in Table 5. The current year’s needles from plants in different treatment groups were collected. The third round of unshaded branches facing south of a single plant from the current year were determined and the needles in the middle of the branches collected. Sampling was carried out from 10:00 to 12:00 on the 90th day of treatment (the end of the experiment).

### 4.4. Index Measurements

#### 4.4.1. Basic Measurements

Needle length was measured using a vernier caliper (precision: 0.01 mm) on current-year needles, which were fully developed by the end of the treatment period. The projected area of two needle fascicles (each containing two needles) was determined using an Epson V700 leaf area scanner. Their fresh weight was recorded using an analytical balance (precision: 0.1 mg). Samples were then oven-dried at 80 °C to constant weight to determine dry weight, and dry matter content was calculated as the ratio of dry weight to fresh weight (g·g^−1^). The specific leaf area (SLA) was calculated by dividing leaf area by dry weight (cm^2^·g^−1^). For anatomical observations, transverse cross-sections of needles were obtained to observe stomatal distribution using a Zeiss Axio Observer fluorescence inverted microscope. Both the adaxial (upper) and abaxial (lower) surfaces of the needles were examined to determine stomatal density, defined as the number of stomata per mm^2^. Leaf width and thickness were measured from these transverse sections. The slicing method involved embedding the needle samples in paraffin, slicing them with a microtome (Leica RM2235), and staining with toluidine blue prior to microscopic imaging [67]. Leaf nitrogen and phosphorus contents were determined using a continuous flow analyzer (Skalar San++ Compact, Skalar Analytical B.V., Breda, Netherlands) after acid digestion with H_2_SO_4_–H_2_O_2_.

#### 4.4.2. Photosynthetic and Chlorophyll Fluorescence Parameters

Measurements of gas exchange parameters were conducted under clear, cloudless skies with stable atmospheric conditions, starting at 09:00 AM local time. Current-year needles from the upper third of the canopy were selected for measurement, and the measurements were conducted immediately at the end of the treatment period. A portable CO_2_/H_2_O infrared gas analyzer (Li-6400, LI-COR, Lincoln, NE, USA) was used. A buffer bottle was installed to ensure that the CO_2_ concentration in the leaf chamber matched ambient levels. The photosynthetically active radiation (PAR) in the chamber was set to 1000 µmol·m^−2^·s^−1^ using a red–blue light source (6400-02B, LI-COR, Lincoln, NE, USA). Before measurements, needles were kept under ambient PAR for at least 15 min to acclimate to the chamber light conditions. The following parameters were recorded: net photosynthetic rate (Pn), stomatal conductance (Gs), intercellular CO_2_ concentration (Ci), and transpiration rate (Tr). Water use efficiency (WUE) was calculated as the ratio of Pn to Tr [27]. The same current-year needles, collected from the same canopy position and at the same time, were used for in vivo chlorophyll fluorescence measurements, conducted using a handheld fluorometer (FluorPen FP110). Prior to measurement, samples were dark-adapted for 30 min using black clips. Initial fluorescence (Fo) and maximum fluorescence (Fm) were recorded to calculate the maximum photochemical efficiency of PSII (Fv/Fm) and the potential photochemical efficiency (Fv/Fo).

#### 4.4.3. Photosynthetic Pigments and Relative Conductivity

Photosynthetic pigment content was determined using the N, N-dimethylformamide method. Current-year needles were collected immediately after the 90-day treatment period from the upper third of the seedling canopy. A 5 g fresh needle sample was fully immersed in N, N-dimethylformamide and incubated in darkness at 4 °C until completely decolorized. Absorbance was measured at 480, 647, and 664 nm using a spectrophotometer, and the contents of chlorophyll a, chlorophyll b, total chlorophyll, and carotenoids were calculated as described by Wellburn (1994) [68]. Relative conductivity was measured using a conductivity meter, following the method described by Lutts et al. (1996) [4], to assess membrane permeability and cell membrane integrity [69].

#### 4.4.4. Protective Enzyme Activity

Fresh leaf samples (0.5 g) were flash-frozen in liquid nitrogen and stored at −80 °C. Activities of superoxide dismutase (SOD) and peroxidase (POD) were subsequently measured [27,67].

#### 4.4.5. Osmoregulatory Substances

Soluble sugar content was determined using the anthrone–sulfuric acid method [70], proline content using the ninhydrin method [71], soluble protein content using the Coomassie Brilliant Blue G-250 staining method [72], and malondialdehyde content using the 2-thiobarbituric acid method [73].

### 4.5. Membership Function Calculation

To comprehensively evaluate the environmental adaptability of different treatments, a membership function method was used. The membership function value is calculated as follows:

Indicators positively correlated with seedling growth:

U(X_i_) = (X_i_ − X_min_)/(X_max_ − X_min_) (1)

Indicators negatively correlated with seedling growth:

U(X_i_)=1 − (X_i_ − X_min_)/(X_max_ − X_min_) (2)

where U is the membership value; i = 1, 2, 3,…, n; X_i_ is the measured value of the indicator; X_min_ is the minimum value of a certain indicator in all treatment groups; and X_max_ is the maximum value of a certain indicator in all treatment groups.

### 4.6. Data Analysis

Data were pre-processed using SPSS 26.0 for the normality and homogeneity of variance tests. A two-way ANOVA was conducted to assess the main and interactive effects of light and nitrogen treatments on all measured traits. Where appropriate, polynomial regression analyses were applied to evaluate quantitative trends. Statistical significance was determined at *p* < 0.05 using SPSS 26.0. Post hoc comparisons were performed using Duncan’s test. Graphs were generated using GraphPad Prism 9.5 and R 4.4.2.

## 5. Conclusions and Prospects

This study systematically evaluated the effects of light–nitrogen interactions on the leaf functional traits of *Picea neoveitchii* Mast. seedlings. The results demonstrated that moderate shading (70% full sunlight) generally enhanced leaf morphological and physiological traits under most nitrogen conditions. In contrast, excessive nitrogen input or low light intensity exerted inhibitory effects, potentially limiting photosynthesis and disturbing metabolic balance, thereby suppressing growth. Membership function analysis identified the L2N0 treatment (70% full sunlight, 0 g N·m^−2^·a^−1^) as the most favorable condition, showing optimal performance in terms of both growth and stress resistance. Future research should explore the long-term effects of light–nitrogen interactions on *Picea neoveitchii* Mast. population dynamics and employ molecular biology techniques to elucidate underlying physiological and metabolic mechanisms, providing a more comprehensive theoretical basis for the conservation and restoration of this endangered species.

## Figures and Tables

**Table 1:** Effects of light and nitrogen interaction on needle anatomical characteristics of *Picea neoveitchii* Mast. seedlings.

Anatomical Characteristics	N Treatment	Shading Treatment		
		L1 100% Full Light	L2 70% Full Light	L3 40% Full Light
Needle thickness (mm)	N0	0.60 ± 0.14 aA	0.79 ± 0.06 aA	0.60 ± 0.03 aA
	N1	0.62 ± 0.14 aA	0.68 ± 0.02 abA	0.55 ± 0.05 aA
	N2	0.66 ± 0.06 aA	0.54 ± 0.11 bA	0.62 ± 0.09 aA
Stomatal density (No. mm^2^)	N0	56.19 ± 20.72 aA	23.32 ± 3.13 bB	31.19 ± 3.53 aB
	N1	33.19 ± 7.36 bA	23.72 ± 3.84 bA	27.95 ± 1.95 aA
	N2	35.82 ± 4.83 abA	37.81 ± 5.22 aA	32.90 ± 3.81 aA
Needle cross-sectional area (10^−2^ mm^2^)	N0	40.01 ± 1.84 aB	81.07 ± 11.60 aA	54 ± 6.67 aB
	N1	49.08 ± 6.34 aA	55.72 ± 6.15 bA	47.6 ± 3.30 aA
	N2	52.33 ± 9.84 aA	50.75 ± 8.19 bA	50.2 ± 2.59 aA
Perimeter of needle section (mm)	N0	2.88 ± 0.47 aA	3.58 ± 0.43 aA	3.01 ± 0.28 aA
	N1	2.78 ± 0.19 aA	2.94 ± 0.16 bA	2.85 ± 0.01 aA
	N2	2.83 ± 0.28 aA	3.1 ± 0.20 abA	2.87 ± 0.17 aA
Epidermal tissue area (10^−2^ mm^2^)	N0	6.03 ± 1.49 aA	8.85 ± 1.81 aA	6.59 ± 0.47 aA
	N1	5.93 ± 1.22 aA	6.49 ± 0.96 aA	6.85 ± 0.57 aA
	N2	7.13 ± 1.44 aA	6.83 ± 0.84 aA	6.51 ± 0.84 aA
Mesophyll tissue area (10^−2^ mm^2^)	N0	30.72 ± 1.73 aB	66.81 ± 9.87 aA	42.3 ± 5.93 aB
	N1	38.12 ± 7.04 aA	45.02 ± 5.32 bA	36.38 ± 3.19 aA
	N2	40.15 ± 7.28 aA	40.25 ± 5.83 bA	39.71 ± 2.25 aA
Resin cavity area (10^−2^ mm^2^)	N0	0.08 ± 0.02 bB	0.16 ± 0.09 aB	0.38 ± 0.13 aA
	N1	0.18 ± 0.04 aA	0.19 ± 0.04 aA	0.26 ± 0.06 abA
	N2	0.18 ± 0.03 aA	0.08 ± 0.02 aA	0.16 ± 0.08 bA
Endocortical area (10^−2^ mm^2^)	N0	1.35 ± 0.15 aB	1.88 ± 0.2 aA	1.56 ± 0.15 aAB
	N1	1.27 ± 0.16 aA	1.49 ± 0.14 abA	1.38 ± 0.15 abA
	N2	1.41 ± 0.27 aA	1.22 ± 0.31 bA	1.20 ± 0.06 bA
Central column area (10^−2^ mm^2^)	N0	2.27 ± 0.20 bB	3.36 ± 0.18 aA	3.29 ± 0.11 aA
	N1	2.53 ± 0.28 bA	2.55 ± 0.24 bA	2.73 ± 0.5 aA
	N2	3.14 ± 0.26 aA	1.88 ± 0.27 cB	2.61 ± 0.35 aA
Mesophyll tissue area ratio (%)	N0	76.76 ± 0.91 bB	82.38 ± 0.47 aA	78.23 ± 1.51 aB
	N1	79.89 ± 1.02 aA	80.76 ± 1.20 abA	76.37 ± 1.35 aB
	N2	76.79 ± 0.59 bA	79.43 ± 1.29 bA	79.11 ± 1.89 aA
Resin cavity area ratio (%)	N0	0.19 ± 0.057 bB	0.19 ± 0.077 bB	0.70 ± 0.149 aA
	N1	0.36 ± 0.10 aA	0.35 ± 0.099 aA	0.55 ± 0.10 abA
	N2	0.35 ± 0.08 aA	0.16 ± 0.017 bB	0.33 ± 0.18 bAB
Area ratio of central column (%)	N0	5.61 ± 0.28 aA	4.21 ± 0.74 aB	6.13 ± 0.59 aA
	N1	5.19 ± 0.51 aAB	4.59 ± 0.36 aB	5.69 ± 0.68 aA
	N2	6.10 ± 0.88 aA	3.71 ± 0.20 aB	5.20 ± 0.57 aA

Different lowercase letters indicate significant differences (*p* < 0.05) between different nitrogen application groups under the same shading treatment; different capital letters indicate significant differences (*p* < 0.05) between different shading treatment groups under the same nitrogen application treatment.

**Table 2:** Effects of light and nitrogen interaction on needle photosynthetic pigments of *Picea neoveitchii* Mast. seedlings.

Photosynthetic Pigment	N Treatment	Shading Treatment		
		L1 100% Full Light	L2 70% Full Light	L3 40% Full Light
Chlorophyll a content (mg·g^−1^)	N0	0.245 ± 0.058 aA	0.162 ± 0.034 aA	0.216 ± 0.03 aA
	N1	0.162 ± 0.027 bA	0.152 ± 0.046 aA	0.127 ± 0.027 bA
	N2	0.143 ± 0.018 bB	0.171 ± 0.036 aB	0.261 ± 0.025 aA
Chlorophyll b content (mg·g^−1^)	N0	0.151 ± 0.042 aA	0.086 ± 0.009 aA	0.124 ± 0.043 bA
	N1	0.093 ± 0.021 bA	0.089 ± 0.041 aA	0.046 ± 0.011 cA
	N2	0.063 ± 0.006 bB	0.092 ± 0.029 aB	0.205 ± 0.033 aA
Total chlorophyll content (mg·g^−1^)	N0	0.396 ± 0.100 aA	0.248 ± 0.043 aB	0.339 ± 0.054 bAB
	N1	0.255 ± 0.048 bA	0.240 ± 0.087 aA	0.173 ± 0.038 cA
	N2	0.206 ± 0.015 bB	0.264 ± 0.065 aB	0.465 ± 0.052 aA
Carotenoid content (mg·g^−1^)	N0	0.061 ± 0.015 aA	0.056 ± 0.005 aA	0.052 ± 0.017 aA
	N1	0.053 ± 0.010 aA	0.044 ± 0.011 aA	0.050 ± 0.010 aA
	N2	0.056 ± 0.004 aA	0.055 ± 0.010 aA	0.038 ± 0.013 aA

Different lowercase letters indicate significant differences (*p* < 0.05) between different nitrogen application groups under the same shading treatment; different capital letters indicate significant differences (*p* < 0.05) between different shading treatment groups under the same nitrogen application treatment.

**Table 3:** Effects of light and nitrogen interaction on needle osmoregulation substance accumulation of *Picea neoveitchii* Mast. seedlings.

Resistance Physiological traits	N Treatment	Shading Treatment		
		L1 100% Full Light	L2 70% Full Light	L3 40% Full Light
Soluble protein content (mg·g^−1^)	N0	1.71 ± 0.27 aA	1.47 ± 0.39 abA	1.44 ± 0.04 aA
	N1	1.68 ± 0.03 aA	1.19 ± 0.11 bB	1.18 ± 0.14 aB
	N2	1.09 ± 0.24 bA	1.73 ± 0.08 aA	1.40 ± 0.47 aA
Free proline content (μg·g^−1^)	N0	304.95 ± 28.67 bA	287.00 ± 22.00 aA	312.84 ± 15.17 bA
	N1	314.56 ± 10.85 bAB	352.51 ± 37.09 aA	281.23 ± 12.48 bB
	N2	445.05 ± 18.41 aA	346.15 ± 55.87 aB	390.19 ± 48.11 aAB
Malondialdehyde content (m·mol g^−1^)	N0	1.3960.815 aA	0.676 ± 0.217 aAB	0.472 ± 0.098 aB
	N1	0.453 ± 0.195 bA	0.550 ± 0.097 aA	0.686 ± 0.355 aA
	N2	0.772 ± 0.337 abA	0.499 ± 0.036 aA	0.970 ± 0.432 aA
Relative electrical conductivity (%)	N0	73.42 ± 3.99 aA	60.66 ± 7.35 aA	64.77 ± 9.97 aA
	N1	63.60 ± 4.80 aA	55.22 ± 3.40 aB	37.00 ± 0.36 bC
	N2	64.60 ± 9.95 aA	38.17 ± 4.17 bB	52.07 ± 4.92 aA
Soluble sugar content (mg·g^−1^)	N0	58.47 ± 6.04 aB	70.50 ± 2.43 aA	48.90 ± 5.99 aB
	N1	65.95 ± 9.43 aA	51.49 ± 6.53 bB	47.19 ± 1.35 aB
	N2	59.17 ± 9.77 aA	55.61 ± 11.89 abA	50.38 ± 14.07 aA
Starch content (mg·g^−1^)	N0	9.99 ± 0.53 bB	11.53 ± 0.97 aAB	11.82 ± 0.76 aA
	N1	9.96 ± 1.56 bA	11.66 ± 1.35 aA	10.01 ± 0.63 bA
	N2	13.92 ± 2.27 aA	12.52 ± 2.43 aAB	9.12 ± 0.98 bB
Unstructured carbohydrates (mg·g^−1^)	N0	68.47 ± 6.49 aB	82.03 ± 1.76 aA	60.71 ± 6.54 aB
	N1	75.91 ± 10.88 aA	63.15 ± 6.14 bAB	57.19 ± 1.82 aB
	N2	73.08 ± 11.88 aA	68.13 ± 13.98 abA	59.5 ± 15.03 aA

Different lowercase letters indicate significant differences (*p* < 0.05) between different nitrogen application groups under the same shading treatment; different capital letters indicate significant differences (*p* < 0.05) between different shading treatment groups under the same nitrogen application treatment.

**Table 4:** Membership function value of leaf functional traits of *Picea neoveitchii* Mast. seedlings.

Index	Subordinate Function Value								
	L1N0	L1N1	L1N2	L2N0	L2N1	L2N2	L3N0	L3N1	L3N2
Needle thickness	0.158	0.445	0.237	0.408	0.746	1.000	0.000	0.685	0.448
Leaf area	0.086	0.610	0.308	0.437	0.824	1.000	0.000	0.360	0.375
Specific leaf area	0.156	0.316	0.000	0.586	1.000	0.754	0.502	0.142	0.345
Leaf dry matter content	0.900	1.000	0.600	1.000	0.000	0.900	0.300	0.600	0.700
Needle thickness	0.260	0.309	0.488	1.000	0.569	0.000	0.248	0.049	0.341
Stomatal density	1.000	0.300	0.380	0.000	0.012	0.441	0.239	0.141	0.291
Needle cross-sectional area	0.000	0.221	0.300	1.000	0.383	0.262	0.341	0.185	0.248
Perimeter of needle section	0.125	0.000	0.063	1.000	0.200	0.400	0.288	0.088	0.113
Epidermal tissue area	0.034	0.000	0.411	1.000	0.192	0.308	0.226	0.315	0.199
Mesophyll tissue area	0.000	0.205	0.261	1.000	0.396	0.264	0.321	0.157	0.249
Resin cavity area	0.000	0.333	0.333	0.267	0.367	0.000	1.000	0.600	0.267
Endocortical area	0.221	0.103	0.309	1.000	0.426	0.029	0.529	0.265	0.000
Central column area	0.264	0.439	0.851	1.000	0.453	0.000	0.953	0.574	0.493
Mesophyll tissue area ratio	0.065	0.586	0.070	1.000	0.730	0.509	0.309	0.000	0.456
Resin cavity area ratio	0.049	0.368	0.344	0.056	0.346	0.000	1.000	0.721	0.314
Area ratio of central column	0.785	0.612	0.988	0.207	0.364	0.000	1.000	0.818	0.616
N content of needles	0.275	0.237	1.000	0.000	0.397	0.971	0.369	0.437	0.724
P content of needles	1.000	0.207	0.272	0.482	0.327	0.267	0.764	0.173	0.000
N/P	1.000	0.671	0.138	0.984	0.620	0.164	0.872	0.404	0.000
Chlorophyll a content	0.881	0.261	0.119	0.261	0.187	0.328	0.664	0.000	1.000
Chlorophyll b content	0.660	0.296	0.107	0.252	0.270	0.289	0.491	0.000	1.000
Total chlorophyll content	0.764	0.281	0.113	0.257	0.229	0.312	0.568	0.000	1.000
Carotenoid content	1.000	0.652	0.783	0.783	0.261	0.739	0.609	0.522	0.000
Net photosynthetic rate	0.260	0.419	0.354	0.574	1.000	0.623	0.000	0.567	0.933
Stomatal conductance	0.056	0.022	0.377	0.242	0.593	0.288	0.000	0.375	1.000
Intercellular CO_2_ concentration	0.303	0.000	0.722	0.443	0.628	0.530	1.000	0.487	0.642
Transpiration rate	0.285	0.170	0.443	0.308	0.364	0.324	0.000	0.611	1.000
Fv/Fm	0.398	0.000	0.496	1.000	0.235	0.656	0.494	0.060	0.438
Fv/Fo	0.237	0.000	0.301	1.000	0.123	0.488	0.313	0.029	0.285
SOD activity	0.890	0.581	0.652	0.824	0.503	1.000	0.000	0.722	0.504
POD activity	1.000	0.581	0.719	0.219	0.909	0.000	0.443	0.456	0.650
CAT activity	0.908	0.832	0.603	0.475	0.168	0.547	0.939	1.000	0.000
Soluble protein content	0.969	0.922	0.000	0.594	0.156	1.000	0.547	0.141	0.484
Free proline content	0.145	0.203	1.000	0.035	0.435	0.396	0.193	0.000	0.665
Malondialdehyde content	0.000	1.000	0.661	0.763	0.897	0.951	0.980	0.752	0.452
Relative electrical conductivity	0.000	0.075	0.040	0.316	0.366	0.611	0.034	1.000	0.476
Soluble sugar content	0.484	0.805	0.514	1.000	0.184	0.361	0.073	0.000	0.137
Starch content	0.181	0.175	1.000	0.502	0.529	0.708	0.563	0.185	0.000
NSC content	0.454	0.754	0.640	1.000	0.240	0.440	0.142	0.000	0.093
Mean	0.409	0.389	0.435	0.592	0.439	0.462	0.433	0.362	0.419
Order	7	8	4	1	3	2	5	9	6

**Table 5:** Double factor combination level in test design.

N Treatment	Shade Levels		
	L1 (100% Full Light)	L2 (70% Full Light)	L3 (40% Full Light)
N0 (0 g m^−2^·a^−1^)	L1N0 (CK)	L2N0	L3N0
N0 (10 g m^−2^·a^−1^)	L1N1	L2N1	L3N1
N1 (20 g m^−2^·a^−1^)	L1N2	L2N2	L3N2

## Data Availability

The data are not publicly available due to privacy or ethical restrictions.

## Supplementary Materials

The following supporting information can be downloaded at: https://www.mdpi.com/article/10.3390/plants14162550/s1, Table S1: Meteorological Data for Photosynthesis Experiments.
